# Restricted Arm Swing in People With Parkinson's Disease Decreases Step Length and Time on Destabilizing Surfaces

**DOI:** 10.3389/fneur.2020.00873

**Published:** 2020-09-25

**Authors:** Tarique Siragy, Mary-Elise MacDonald, Julie Nantel

**Affiliations:** School of Human Kinetics, University of Ottawa, Ottawa, ON, Canada

**Keywords:** dynamic stability, walking stability, arm swing, perturbation, uneven terrain

## Abstract

**Introduction:** Fall rates in people with Parkinson's Disease range between 35 and 68% with the majority of falls occurring while walking. Initial evidence suggests that when walking without arm swing, people with Parkinson's Disease adapt their stepping foot placement as a means to preserve dynamic stability. However, it remains unexamined what arm swing's effect has on dynamic stability when walking on destabilizing surfaces.

**Methods:** Twenty people with Parkinson's Disease (63.78 ± 8.97 years) walked with restricted and unrestricted arm swing on unperturbed, rocky, rolling-hills, and mediolateral translational surfaces. Data were collected on a split-belt treadmill CAREN Extended-System (Motek Medical, Amsterdam, NL). Bilateral averages and coefficient of variations for step time, length, and width; and mediolateral margin of stability were calculated.

**Results:** Results were examined in three separate analyses that included arm conditions during each of the destabilizing surfaces compared to unperturbed walking (arm-rolling hills, arm-rocky, and arm-mediolateral). Compared to unrestricted arm swing, restricted arm swing reduced average step length (arm-rolling hills) and time (arm-rocky), and increased COV step time (arm-rolling hills). The arm-rolling hills analysis revealed that the most affected leg had a shorter step length than the least affected. The destabilizing surface effects revealed that during the arm-rolling hills and arm-rocky analyses, step time decreased, step width increased, and the COV for step time, length and width increased. No main effects occurred for the arm-mediolateral analysis.

**Conclusion:** Results indicate that foot placement in response to restricted arm swing, in people with Parkinson's Disease, depends on the encountered destabilizing surface. The arm-rolling hills analysis revealed that participants appropriately reduced step length as compensation to their restricted arm swing. However, the arm-rocky analysis revealed that individuals prioritized forward progression over dynamic stability as they decreased average step time. Additionally, the increased spatiotemporal variability in response to the rocky and rolling hills conditions indicate partial foot placement adaptation to maintain an already existing level of global dynamic stability as no changes in the Margin of Stability occurred. Adaptation is further corroborated by the decreased step time and increased step width. These responses reflect attempts to pass the destabilizing terrains faster while increasing their base of support.

## Introduction

Fall rates in people with Parkinson's Disease range between 35 and 68% during a 12-months period, with the majority of falls occurring during walking (1–3). These numbers are alarming as falling is closely associated with severe medical and socioeconomic consequences which hold lasting impacts on quality of life (1, 2).

The dynamical nature of gait increases fall risk. Indeed, during the swing phase, the center of mass (COM) is outside the base of support (BOS) (4). Thus, the neuromuscular system can only achieve stability by accurately predicting the future position of the COM in order to correctly place the foot at ground contact (4). Additionally, even during double-support when the COM is within the BOS, stability is challenged as the velocity of the COM is redirected laterally from the unloading to the loading leg (5). As the stepping foot's placement in both the anteroposterior and mediolateral directions is determined by the COM's trajectory; the neuromuscular system strives to maintain a sinusoidal trajectory while it is volitionally displaced inside and outside the BOS (dynamic stability) (4, 5). Disruptions to this trajectory would either require corrective stepping or result in a fall (4, 5). For instance, in people with Parkinson's Disease, impairments to postural control and gait threatens their ability to maintain the COM along a stable trajectory thereby heightening their fall risk (6–10). Further, their balance in the mediolateral direction is particularly affected as posturography studies demonstrate that people with Parkinson's Disease have greater trunk sway in this direction than healthy elderly adults (11, 12). This is particularly concerning as impaired mediolateral balance is predictive of falls, closely associated with hip fractures, and increased mortality rates (13, 14).

Classically, gait paradigms in people with Parkinson's Disease are based on the inverted pendulum model (15, 16). This model proposes that arm swing only has a minimal impact on COM motion since the head, arms and trunk are considered a single nearly-rigid body (16). While arm swing's effect on dynamic stability in healthy adults demonstrates no differences between constrained and unconstrained arm swing, initial evidence suggests that walking without arm swing during unperturbed conditions in people with Parkinson's Disease has a detrimental effect on their dynamic stability (16–20). Siragy and Nantel found that when arm swing was experimentally constrained, people with Parkinson's Disease increased the distance of their COM's dynamical state (position and velocity) to the edges of their BOS compared to unconstrained arm swing conditions (17). The authors suggested that this was a compensatory mechanism to mitigate destabilization caused by an observed increase in trunk angular velocity during their constrained arm swing trials (17).

Thus, arm swing may hold additional implications for dynamic stability in people with Parkinson's Disease when ambulating on challenging surfaces that mechanically perturb gait. Destabilizing surfaces such as rocky, rolling hills, and mediolateral translations are common terrains used in virtual reality gait paradigms to simulate everyday real-world surfaces that destabilize the COM's trajectory during walking (21, 22). Indeed, rocky terrains simulate walking on rocky surfaces, rolling hills simulate a forest trail, and the mediolateral translational simulates walking on a train or a bus (21). When walking on these terrains, healthy young adults adapt their gait specifically to each surface as each terrain has distinct destabilizing effects on the COM's trajectory due to perturbation type and movement direction (21–24). Determining how individuals with Parkinson's disease ambulate on destabilizing surfaces that emulate real-world terrains will provide a greater depiction of how these individuals walk in their daily lives. Further, as arm swing becomes reduced and ultimately absent with disease progression, determining its effect on dynamic stability in people with Parkinson's Disease while on these surfaces holds direct implications for fall prevention (25).

Therefore, the purpose of this research is to examine unrestricted and restricted arm swing's effect on dynamic stability in people with Parkinson's Disease when walking on destabilizing surfaces (rocky, rolling hills, and mediolateral translational). We hypothesize that restricted arm swing will be more unstable, compared to unrestricted, for all terrains and that each terrain will be more unstable than steady-state walking. Additionally, we hypothesize an interaction where participants will have a larger step width during restricted arm swing on the destabilizing surface compared to unrestricted arm swing on the same surface. However, no step width difference will occur between unrestricted and restricted arm swing during unperturbed walking conditions.

## Methods

### Participants

A convenience sample of 20 people with Parkinson's Disease (13 males; seven females), aged 48–79 years old (63.78 ± 8.97 years) were recruited from the Ottawa-Gatineau community. However, as two participants presented severe dyskinesia and two had missing data, only 16 participants were included in the final analysis. Participants were assessed with the original Unified Parkinson's Disease Rating Scale Motor examination (11 ± 6) and were between I-III on the Hoehn and Yahr scale (26, 27). Additionally, motor asymmetry to determine the least and most affected sides was determined with the UPDRS and by participants self-reporting laterality. Average disease duration (8.0 ± 5.1 years) and age at onset (56.8 ± 9.60 years) data were collected. Further, seven participants reported freezing of gait based on the Freezing of Gait Questionnaire. Participants were tested on their optimally medicated state. Prior to data collection, volunteers were excluded if they reported any physical discomfort using a virtual reality system, reported any injuries and/or orthopedic surgeries that could interfere with gait, could walk only with the use of a walking aid, and had any additional illnesses other than Parkinson's Disease. All participants provided written informed consent and the study was approved by both the Hospital and University ethics and scientific committees.

### Procedures

Data collection was conducted as part of a larger protocol that examined the effects of unrestricted and restricted arm swing conditions in people with Parkinson's Disease during unperturbed and perturbed walking conditions (17). This article examines the differences between restricted and unrestricted arm swing conditions during the unperturbed and destabilizing surface trials within this protocol. Participants walked with two arm conditions (restricted and unrestricted) on unperturbed and destabilizing surfaces. Restricted arm swing trials were conducted by participants inserting their arms into their safety harness. This safety harness was worn at all times and attached to an overhead structure to ensure participant safety. The two unperturbed surface trials, one per arm swing condition, lasted 3 min each and included steady-state treadmill walking. During these trials, participants walked through a virtual park environment that had its optical flow synchronized to the treadmill. For the destabilizing surface trials, participants completed two walking trials, one per arm swing condition. During the destabilizing surfaces trials, participants also walked through a virtual park environment, which included three different destabilizing surfaces (rocky, rolling hills, and mediolateral translational) (22). The rocky terrain was simulated with a pseudorandom perturbation in three different axes (vertical, pitch, and roll), rolling hills with an anteroposterior rotational perturbation, and the mediolateral translational was a lateral platform translation (22). All terrain magnitudes and specifications are depicted in Table 1. Terrain specifications were replicated from previously published protocols examining gait on destabilizing surfaces in individuals with lower limb amputation (22). The optical flow during destabilizing surface trials was tied to the treadmill. Specifically, the virtual park environment displayed a park terrain where participants walked along a virtual trail. The trail lasted a simulated 200 m in total and was divided into flat unperturbed segments and the three destabilizing surfaces. Within the 200 m, participants encountered all three destabilizing surfaces that were presented in a pseudorandom order. Each surface lasted 20 m and were separated from each other by 40 m of flat unperturbed walking. When participants encountered one of the destabilizing surfaces, the projection for the park trail changed to mirror the surface condition. For instance, the trail for the rocky surface segment was a visual simulation of rocks appearing on the simulated pathway, the rolling-hills segment was a series of small incline and decline slopes in the pathway, and the mediolateral translation was a projection of a jagged pathway. No visual perturbations nor distortions to the participants' optical field occurred. For all trials, participants walked at preferred walking speeds with both belts running symmetrically. Trial order was randomized per block (unperturbed and destabilizing surfaces). Participants were provided with rest time, both between trials and within the larger protocol, for as long as they desired in order to minimize fatigue.

**Table 1:** Destabilizing surfaces descriptions within the CAREN-Extended System.

**Terrain**	**Description**
Rocky	The CAREN Rumble module causes the platform to oscillate simultaneously in three directions. There was a maximum range of ±2 cm at 0.6Hz vertically, ±1 degree at 1Hz pitch, and ±1 degree at 1.2Hz roll (22).
Rolling-hills	In the AP direction, the platform oscillates based on a sum of four sines at 0.16, 0.21, 0.24, and 0.49Hz. The maximum range was ±3 degrees based on an amplitude scaling of A_w_ = 0.01 (22).
Mediolateral translational	The platform mediolaterally oscillates based on a sum of four sines at 0.16, 0.21, 0.24, and 0.49Hz. The maximum range was ±4 cm based on amplitude scaling of A_w_ = 0.015 (22).

3D motion analysis was completed using the CAREN-Extended System (Motek Medical, Amsterdam NL) using the virtual park environment setting. This system combines a six degrees-of-freedom motion platform with embedded dual-belt treadmill, 12 camera Vicon motion capture system, 180-degree projector screen for virtual world projection, and a safety harness. Three markers placed in the periphery of the treadmill tracked platform motion along with a 57-marker set for tracking full body kinematics (17–19, 22).

### Data Analysis

Markers and ground reaction forces (GRF) were processed in Vicon Nexus (Nexus 2.6, Oxford, UK), while 3D kinematics and kinetics were calculated in Visual 3D. A 4th-order, low-pass, Butterworth filter with a 12 Hz cut-off frequency was used to filter marker data. To remove start-up effects, the first 25 s were removed from trials before data analysis. As each destabilizing surface had an average of 20 steps, 20 consecutive steps were taken at random from the unperturbed trials for all analyses. Data were further analyzed in custom Matlab scripts (Mathworks, Natick, MA) to calculate average spatiotemporal parameters (step time, length, and width) and dynamic stability measures including the Coefficient of Variation (COV) and Margin of Stability (MOS) using previously reported methods (17, 18, 28–30).

The COV was calculated (standard deviation/average ×100) for step time, length and width. The MOS was calculated bilaterally at both heel strikes and defined as the distance of the Extrapolated Center of Mass (xCOM) to the right/left lateral heel marker.

$$\begin{array}{l} MOS=Lateral\;Heel\;Marker-xCOM \end{array}$$

The formula for xCOM was:

$$xCOM=COMp\;+\;(\frac{COMv}{\omega \theta })$$

Where COM_p_ = COM's position, COM_v_ = COM's velocity. ω_θ_ was calculated as:

ωθ=√g/l

In this term, *g* = 9.81 m/s^2^ and *l* is the length of the inverted pendulum determined as the average distance of the right/left lateral heel marker to the COM at heel-strikes. Visual 3D was used to calculate the COM's position and velocity. The MOS was only calculated in the mediolateral direction as this metric is only valid in this direction during walking (31). Visual 3D was used to calculate the COM's position and velocity.

### Statistical Analysis

Data were analyzed in SPSS version 26 and *p* < 0.05 was considered statistically significant. Normality of variables was verified using Shapiro-Wilks tests and three separate three-way repeated measures ANOVA were performed to find the effect of arm swing (restricted and unrestricted), surface (unperturbed and destabilizing surfaces), leg (most and least affected), and potential interactions. Walking speed between trials were assessed with a paired samples *t*-test. If statistical significance was found for preferred walking speed, a General Linear Model with speed as a covariate was performed. Pairwise comparisons with Sidak-Bonferroni corrections were used for *post-hoc* analyses.

## Results

**Figure 1:**
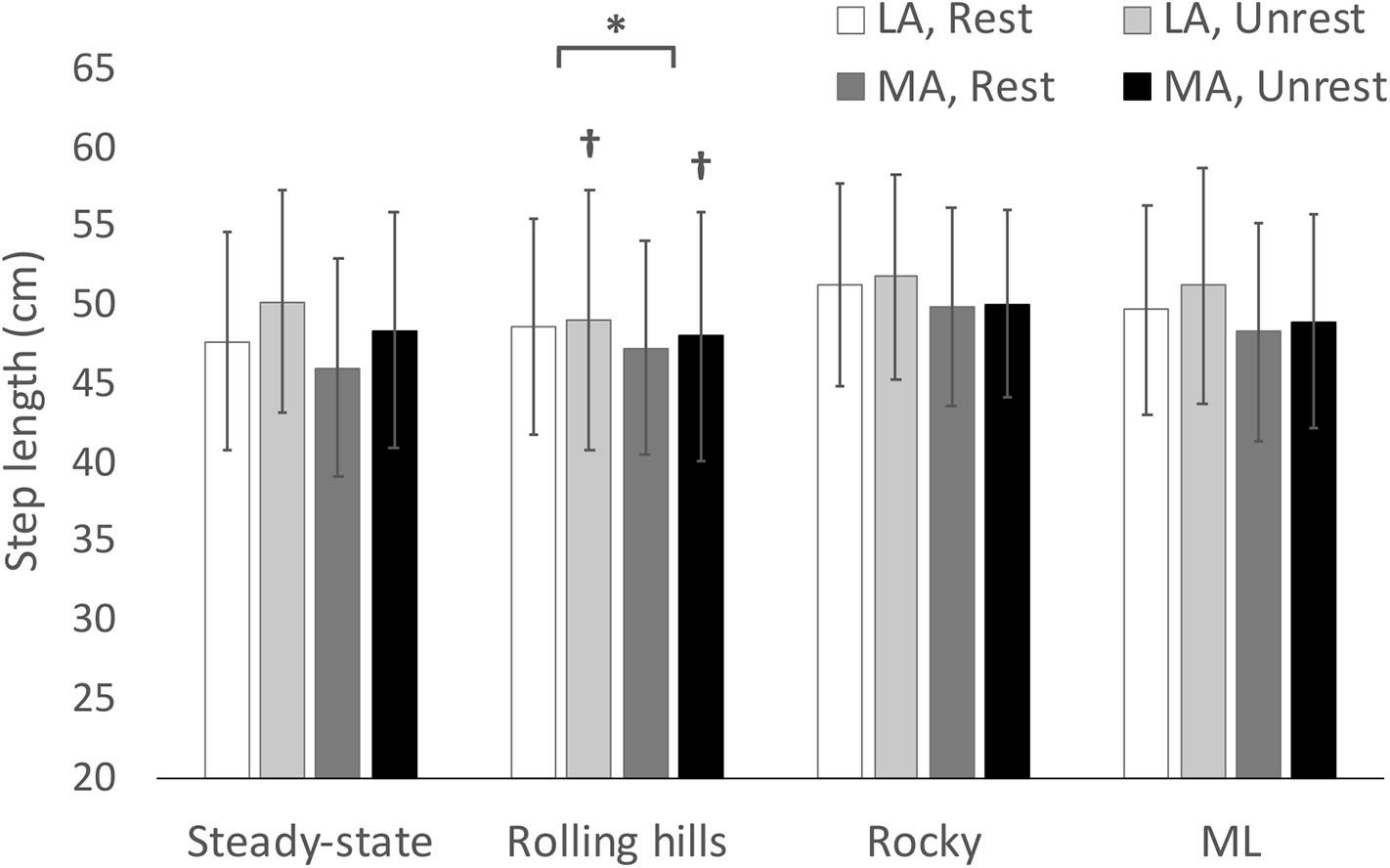
Average step length with respect to terrain conditions for most (MA) and least affected (LA) leg in both restricted and unrestricted arm swing conditions. †represents a significantly larger value in the unrestricted arm swing condition than restricted within the same leg and terrain condition *p* < 0.05. *represents a significantly larger value in LA than MA within the same terrain at *p* < 0.05.

**Figure 2:**
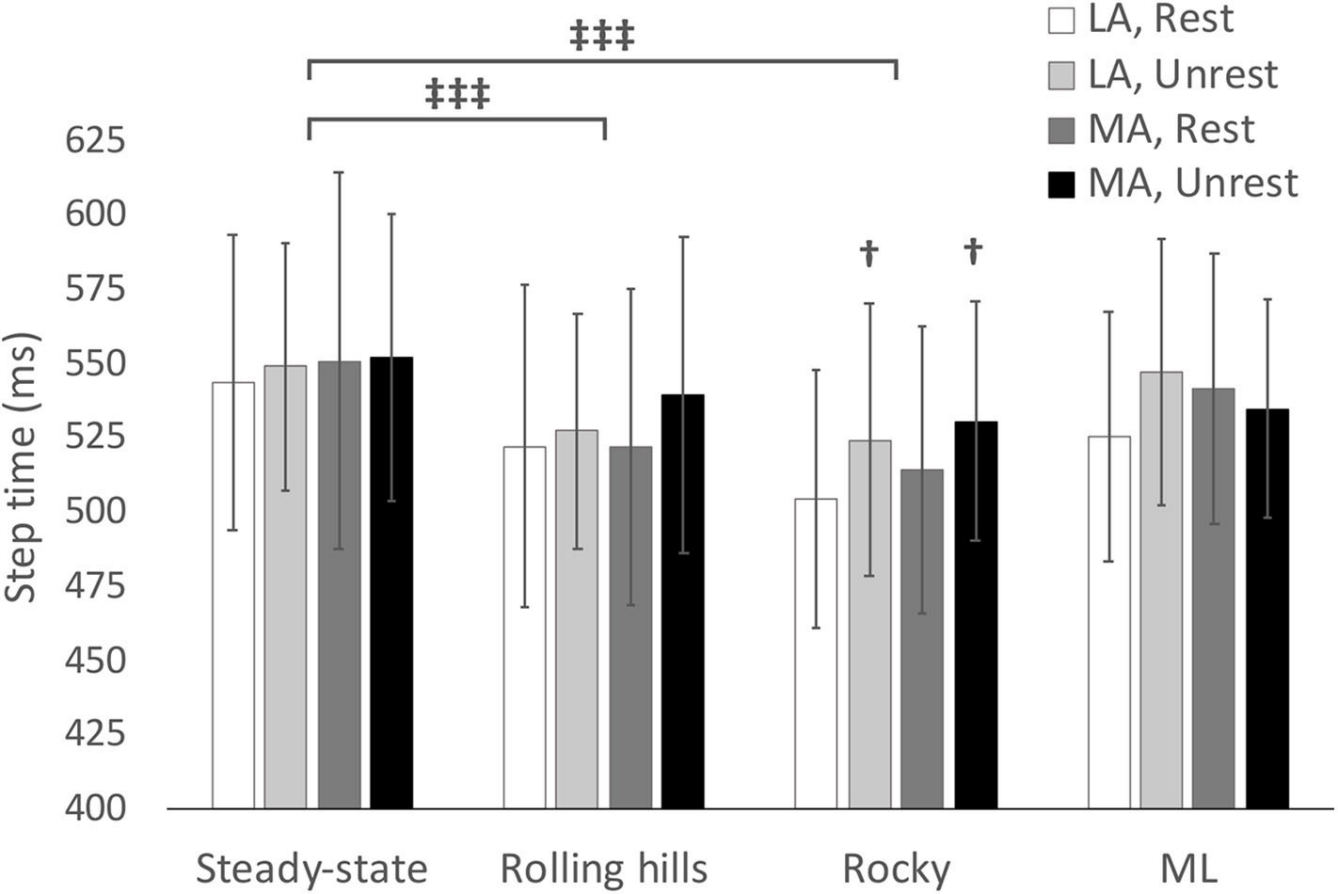
Average step time with respect to terrain conditions for most (MA) and least affected (LA) leg in both restricted and unrestricted arm swing conditions. †represents significantly larger value in the unrestricted arm swing condition than restricted within the same leg and terrain condition *p* < 0.05. represents a significant difference between terrain conditions at *p* < 0.05.

**Figure 3:**
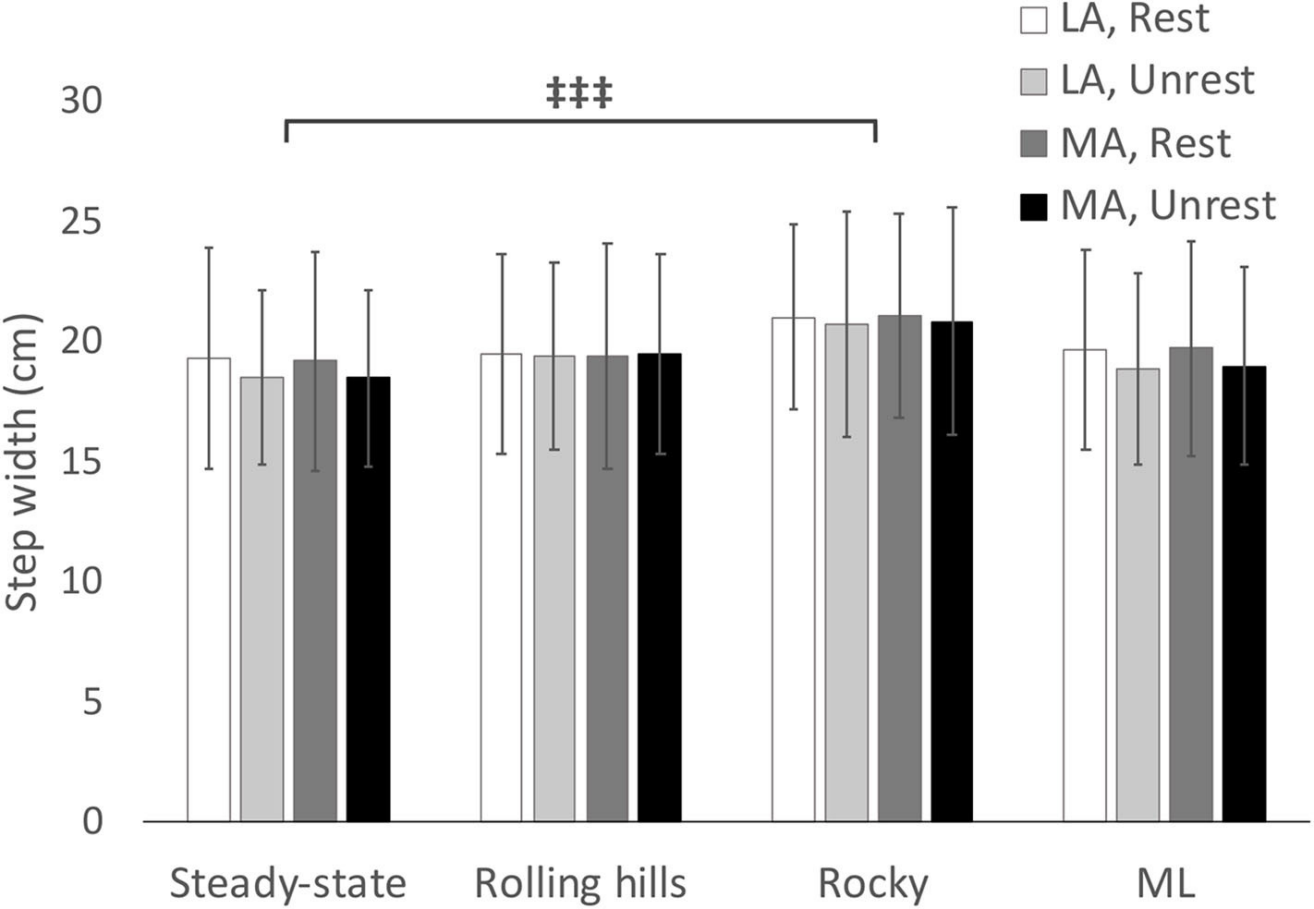
Average step width with respect to terrain conditions for most (MA) and least affected (LA) leg in both restricted and unrestricted arm swing conditions. represents a significant difference between terrain conditions at *p* < 0.05.

Descriptive statistics for dynamic stability measures (COV and MOS) are reported in Table 2A (Arm-Rolling Hills), Table 2B (Arm-Rocky), and Table 2C (Arm-ML Translational). Averages for step length, time and width are reported in Figures 1–3, respectively. Results are reported bilaterally for least and most affected legs. Average walking speeds for each condition are included in Table 3. Since no difference between arm swing conditions within each terrain were found (*p* > 0.05) the two conditions for each terrain were averaged for assessing differences between unperturbed walking and each destabilizing surface.

**Table 2A:** Dynamic Stability Measures for arm (unrestricted and restricted) and terrain (steady-state and rolling hills) conditions: Margin of Stability and Coefficient of Variation for Step Time, Length, and Width along with spatiotemporal averages.

	**Leg**	**Unrestricted**		**Restricted**		***P*-value**
		**Steady-state**	**Rolling hills**	**Steady-state**	**Rolling hills**	
Margin of Stability (cm)	Least	11.30 ± 1.1	11.46 ± 1.5	11.80 ± 1.6	11.39 ± 1.9	0.086
	Most	10.96 ± 1.8	11.02 ± 2.4	11.36 ± 2.0	11.62 ± 2.4	
COV Step Length^*^	Least	3.02 ± 1.1	7.09 ± 2.1	4.41 ± 2.7	7.61 ± 2.2	0.169
	Most	3.79 ± 1.4	7.04 ± 3.2	4.18 ± 2.2	7.01 ± 1.8	
SD Step Length (cm)^*^	Least	1.5 ± 0.4	3.6 ± 1.2	2.0 ± 1.2	3.8 ± 1.2	0.295
	Most	1.8 ± 0.5	3.4 ± 1.7	2.0 ± 1.0	3.3 ± 0.8	
COV Step Time^*†^	Least	2.56 ± 0.5	5.71 ± 2.4	2.86 ± 1.5	6.45 ± 2.3	0.027
	Most	2.37 ± 0.6	6.12 ± 2.0	3.26 ± 2.0	7.00 ± 2.4	
SD Step Time (ms)^*†^	Least	14 ± 3	30 ± 12	16 ± 8	33 ± 9	0.041
	Most	13 ± 3	33 ± 11	18 ± 12	36 ± 11	
COV Step Width^*^	Least	8.75 ± 3.2	11.16 ± 3.5	7.21 ± 3.4	10.41 ± 4.7	0.156
	Most	7.91 ± 2.7	10.79 ± 3.9	7.31 ± 2.9	10.59 ± 4.1	
SD Step Width (cm)^*^	Least	1.6 ± 0.6	2.1 ± 0.6	1.3 ± 0.5	2.0 ± 0.7	0.171
	Most	1.4 ± 0.5	2.0 ± 0.6	1.3 ± 0.3	2.1 ± 1.0	

*Values are reported for the least and most affected legs. P-values from the three-way repeated measures ANOVA are reported for arm swing main effects*.

*Arm Swing Main Effects at p < 0.05*.

* *Terrain Main Effects at p < 0.05*.

**Table 2B:** Dynamic Stability Measures for arm (unrestricted and restricted) and terrain (steady-state and rocky) conditions: Margin of Stability and Coefficient of Variation and Standard Deviation for Step Time, Length, and Width.

	**Leg**	**Unrestricted**		**Restricted**		***P*-value**
		**Steady-state**	**Rocky**	**Steady-state**	**Rocky**	
Margin of Stability (cm)	Least	11.30 ± 1.1	11.79 ± 1.5	11.80 ± 1.6	11.66 ± 1.2	0.127
	Most	10.96 ± 1.8	10.64 ± 2.4	11.36 ± 2.0	11.22 ± 1.9	
COV Step Length^*^	Least	3.02 ± 1.1	7.59 ± 2.8	4.41 ± 2.7	7.63 ± 3.4	0.613
	Most	3.79 ± 1.4	8.15 ± 3.0	4.18 ± 2.2	7.44 ± 2.0	
SD Step Length (cm)^*^	Least	1.5 ± 0.4	3.6 ± 1.1	2.0 ± 1.2	3.6 ± 1.4	0.687
	Most	1.8 ± 0.5	3.8 ± 1.2	2.0 ± 1.0	3.5 ± 0.8	
COV Step Time^*^	Least	2.56 ± 0.51	6.26 ± 2.0	2.86 ± 1.5	6.80 ± 1.8	0.457
	Most	2.37 ± 0.56	6.82 ± 2.4	3.26 ± 2.0	6.19 ± 1.9	
SD Step Time (ms)^*^	Least	14 ± 3	33 ± 10	16 ± 8	34 ± 8	0.625
	Most	13 ± 3	36 ± 12	18 ± 12	32 ± 10	
COV Step Width^*^	Least	8.75 ± 3.2	15.63 ± 9.9	7.21 ± 3.4	13.73 ± 5.5	0.095
	Most	7.91 ± 2.7	14.93 ± 8.5	7.31 ± 2.9	14.25 ± 7.3	
SD Step Width (cm)^*^	Least	1.6 ± 0.6	2.9 ± 0.9	1.3 ± 0.5	2.8 ± 0.8	0.207
	Most	1.4 ± 0.5	2.9 ± 1.0	1.3 ± 0.3	2.8 ± 0.8	

*Values are reported for the least and most affected legs. P-values from the three-way repeated measures ANOVA are reported for arm swing main effects*.

*Arm Swing Main Effects at p < 0.05*.

* *Terrain Main Effects at p < 0.05*.

**Table 2C:** Dynamic Stability Measures for arm (unrestricted and restricted) and terrain (steady-state and mediolateral translational) conditions: Margin of Stability and Coefficient of Variation for Step Time, Length, and Width along with spatiotemporal averages.

	**Leg**	**Unrestricted**		**Restricted**		***P*-value**
		**Steady-state**	**Mediolateral**	**Steady-state**	**Mediolateral**	
Margin of stability (cm)	Least	11.23 ± 1.2	11.03 ± 1.5	11.78 ± 1.7	11.36 ± 1.8	0.442
	Most	10.83 ± 1.8	10.81 ± 2.2	11.20 ± 2.0	11.05 ± 2.1	
COV step length	Least	3.02 ± 1.1	4.46 ± 1.6	4.41 ± 2.7	5.11 ± 1.9	0.492
	Most	3.79 ± 1.4	4.82 ± 1.5	4.18 ± 2.2	5.26 ± 1.7	
SD step length (cm)	Least	1.5 ± 0.4	2.3 ± 0.9	2.0 ± 1.2	2.6 ± 0.9	0.451
	Most	1.8 ± 0.5	2.4 ± 0.7	2.0 ± 1.0	2.6 ± 0.9	
COV step time	Least	2.56 ± 0.51	3.94 ± 2.2	2.86 ± 1.5	3.78 ± 2.5	0.928
	Most	2.37 ± 0.56	4.05 ± 1.6	3.26 ± 2.0	4.29 ± 2.0	
SD step time (ms)	Least	14 ± 3	21 ± 11	16 ± 8	20 ± 13	0.992
	Most	13 ± 3	22 ± 8	18 ± 12	23 ± 9	
COV step width	Least	8.75 ± 3.2	16.27 ± 7.5	7.21 ± 3.4	15.06 ± 8.4	0.638
	Most	7.91 ± 2.7	16.29 ± 6.4	7.31 ± 2.9	15.97 ± 9.7	
SD step width (cm)	Least	1.6 ± 0.6	2.9 ± 1.0	1.3 ± 0.5	2.7 ± 1.0	0.809
	Most	1.4 ± 05	2.9 ± 0.8	1.3 ± 0.3	2.9 ± 1.3	

*Values are reported for the least and most affected legs. P-values from the three-way repeated measures ANOVA are reported for arm swing main effects*.

*Arm Swing Main Effects at p < 0.05*.

* *Terrain Main Effects at p < 0.05*.

**Table 3:** Average walking speed and standard deviation for each walking trial for arm swing conditions and destabilizing surfaces.

**Condition**	**Average walking speed (m/s)**
Unperturbed unrestricted arm swing	0.99 ± 0.16
Unperturbed restricted arm swing	0.96 ± 0.17
Rolling hills unrestricted arm swing	1.03 ± 0.13
Rolling hills restricted arm swing	1.03 ± 0.14
Rocky unrestricted arm swing	1.04 ± 0.14
Rocky restricted arm swing	1.03 ± 0.14
Mediolateral translational unrestricted arm swing	1.04 ± 0.14
Mediolateral translational restricted arm swing	1.05 ± 0.14

### Rolling Hills

An arm swing main effect occurred whereby restricted arm swing reduced step length [*F*_(1, 15)_ = 5.86, *p* = 0.029, $\eta_{\text{p}}^{2}$ = 0.281], increased COV step time [*F*_(1, 15)_ = 5.98, *p* = 0.027, $\eta_{\text{p}}^{2}$ = 0.285], and increased Step Time Standard Deviation (SD) [*F*_(1, 15)_ = 5.00, *p* = 0.041, $\eta_{\text{p}}^{2}$ = 0.250]. The ANOVA further revealed a terrain main effect where the rolling hills surface, compared to unperturbed walking, had a reduced average step time [*F*_(1, 15)_ = 6.11, *p* =0.026, $\eta_{\text{p}}^{2}$ = 0.289], increased COV step length [*F*_(1, 15)_ = 55.62, *p* < 0.001, $\eta_{\text{p}}^{2}$ = 0.788], increased Step Length SD [*F*_(1, 15)_ = 65.98, *p* < 0.001, $\eta_{\text{p}}^{2}$ = 0.815], increased COV step time [*F*_(1, 15)_ = 57.58, *p* < 0.001, $\eta_{\text{p}}^{2}$ = 0.793], increased Step Time SD [*F*_(1, 15)_ = 57.89, *p* < 0.001, $\eta_{\text{p}}^{2}$ = 0.794], increased COV step width [*F*_(1, 15)_ = 21.49, *p* < 0.001, $\eta_{\text{p}}^{2}$ = 0.589] and increased Step Width SD [*F*_(1, 15)_ = 29.10, *p* < 0.001, $\eta_{\text{p}}^{2}$ = 0.660]. Additionally, a leg main effect was observed where the most affected leg had a reduced step length compared to the least affected [*F*_(1, 15)_ = 5.42, *p* = 0.034, $\eta_{\text{p}}^{2}$ = 0.265]. No further main effects or interactions were found.

### Rocky

The ANOVA demonstrated an arm swing main effect in that the restricted arm swing had a reduced average step time [*F*_(1, 15)_ = 6.712, *p* = 0.020, $\eta_{\text{p}}^{2}$ = 0.309]. Further, the ANOVA revealed a surface main effect in that the rocky surface, compared to unperturbed walking, had a reduced average step time [*F*_(1, 15)_ = 14.00, *p* = 0.002, $\eta_{\text{p}}^{2}$ = 0.483], increased average step width [*F*_(1, 15)_ = 12.24, *p* = 0.003, $\eta_{\text{p}}^{2}$ = 0.449], increased COV Step Length [*F*_(1, 15)_ = 52.27, *p* < 0.001, $\eta_{\text{p}}^{2}$ = 0.777], increased Step Length SD [*F*_(1, 15)_ = 92.21, *p* < 0.001, $\eta_{\text{p}}^{2}$ = 0.860], increased COV step time [*F*_(1, 15)_ = 88.56, *p* < 0.001, $\eta_{\text{p}}^{2}$ = 0.856], increased Step Time SD [*F*_(1, 15)_ = 83.89, *p* < 0.001, $\eta_{\text{p}}^{2}$ = 0.848], increased COV step width [*F*_(1, 15)_ = 16.38, *p* < 0.001, $\eta_{\text{p}}^{2}$ = 0.522], and an increased Step Width SD [*F*_(1, 15)_ = 61.99, *p* < 0.001, $\eta_{\text{p}}^{2}$ = 0.805]. An interaction for COV step time occurred for arm, leg, and surface [*F*_(1, 15)_ = 5.29, *p* = 0.036, $\eta_{\text{p}}^{2}$ = 0.261], however, the *post-hoc* revealed no significant differences. No additional main effects or interactions were found.

### Mediolateral Translational

As speed was statistically significant between unperturbed walking trials and the mediolateral translational surface [*t*_(1, 15)_ = −2.445, *p* = 0.028], the General Linear Model with speed as a covariate was used for this analysis. This analysis revealed no main effects for arm or terrain (*p* > 0.05). Additionally, a leg main effect occurred for average step width [*F*_(1, 15)_ = 4.97, *p* = 0.045, $\eta_{\text{p}}^{2}$ = 0.274], however, the *post-hoc* comparison was non-significant.

## Discussion

This study examined how arm swing (restricted and unrestricted) and destabilizing surfaces (rocky, rolling hills, and mediolateral translational) affected dynamic stability in people with Parkinson's Disease. The results support our hypothesis that restricted arm swing was more destabilizing than unrestricted arm swing and that the rocky and rolling hills surfaces were more destabilizing than unperturbed treadmill walking. However, both dynamic stability measures (COV and MOS) responded uniquely to both arm and surface conditions. For instance, COV metrics increased during both arm and surface conditions yet no changes occurred in the MOS. Interestingly, a leg main effect was observed during the arms-rolling hills analysis where step length was shorter in the most affected leg compared to the least affected. Unexpectedly, no difference occurred between the mediolateral translational surface and unperturbed walking.

### Arm Swing

Our results demonstrated that restricted arm swing reduced average step length (arm-rolling hills) and time (arm-rocky) while increasing variability for step time (arm-rolling hills) compared to the unrestricted arm swing condition. Previous research demonstrates that when people with Parkinson's Disease walk with restricted arm swing, compared to unrestricted, trunk angular velocity about the vertical axis increases (17). During walking, a nominal 1:1 contralateral arm-leg swing controls trunk angular motion about the vertical axis by equalizing torques acting on the COM (16). However, when arm swing is restricted, the torques arising from the leg act on the COM unattenuated. This in turn causes the trunk to rotate faster as no counterbalancing torque from the arms are present to mitigate the torques from the legs. In people with Parkinson's Disease, the increased trunk rotation consequently causes the COM's average angular velocity about the vertical axis to increase (17). This increase in angular velocity subsequently acts as an internal perturbation as it increases spatiotemporal variability in these individuals (17). As foot placement at heel-strike is based on the COM's predicted trajectory, the increased variability indicates that the COM is moving along a more variable trajectory. In response to this increased velocity, people with Parkinson's Disease adopt a trunk stiffening strategy and adapt the magnitude of their foot placement (17). Both of which are suggested to be compensatory responses to reduce forward balance loss during heel-strike when the COM begins to transfer from the unloading to the loading leg (17).

For instance, reductions in step length, during restricted compared to unrestricted arm swing conditions, are suggested to attenuate simultaneous increases in trunk angular velocity about the vertical axis, in lieu of the contralateral arm-leg swing pattern (17). Indeed, in an examination of step length amplitudes, Huang et al. demonstrated that reductions in step length were accompanied by simultaneous reductions in spinal rotation amplitudes (32). As such, the reduced step length in our study appears to be a strategy to control internal destabilization arising from excessive trunk rotation when arm swing is restricted. Contrastingly, the increased COV step time in the arm-rolling hills analysis could be more indicative of motor impairment than adaptation. Indeed, in people with Parkinson's Disease, neurodegeneration in the Basal Ganglia impairs rhythmic internal movement timing (33). Similar findings were reported by Siragy and Nantel in an assessment of restricted arm swing and unperturbed walking in this demographic (17). The authors proposed that the increase in trunk angular velocity about the vertical axis, during the restricted arm swing condition, disrupts the rhythmic temporal sequence of foot placement (17). This holds direct implications for clinicians as COV step time is a strong predictor of falls in this demographic (6, 34). Specifically, as arm swing becomes completely absent as the disease progresses, the internal timing of foot placement during walking is disrupted by both the increases in trunk rotation as well as the continued neurodegeneration of the disease. Based on this evidence, future research should consider examining the effect of mechanically restoring the contralateral arm-leg swing pattern in people with Parkinson's Disease as an intervention to facilitate rhythmic gait.

Interestingly, the arms-rolling hills analysis further revealed that the most affected leg had a reduced average step length compared to the least affected leg. In Parkinson's Disease, one of the primary symptoms of the disease is the reduced movement amplitude (hypokinesia) that occurs when individuals are performing a motor task (33, 35–37). In gait, one of the main manifestations of hypokinesia is the reduced step length exhibited by this demographic compared to healthy elderly adults (33, 37). However, in the early to moderate stages of the disease, the neurodegeneration is asymmetric, thereby causing the symptomatology to be expressed more in one limb than the other (38, 39). Thus, step length in people with Parkinson's Disease is not only reduced in amplitude but is also asymmetric compared to healthy elderly adults (8, 9). However, it is interesting to note that the step length interlimb differences in our study only occurred during the arms-rolling hills analysis. This is likely due to the distinct perturbing effects that different destabilizing surfaces have on gait (21–24). In our study, the main destabilization that arose from the rolling hills surface acted in the anteroposterior direction. As such, differences between limbs in anteroposterior foot placement may have only been elicited when a mechanical perturbation acts in the same direction.

Similarly, the current findings also suggest that the effect of restricted arm swing on our participants varied based on the specific terrain encountered. Indeed, unlike the results from the arm-rolling hills analysis, the arm-rocky analysis revealed a reduction in average step time for the restricted arm swing condition. This finding was unexpected as individuals typically increase step time when dynamic stability is threatened as part of the “cautious gait” strategy (40). However, during unperturbed walking, people with Parkinson's Disease increase their cadence, relative to healthy aged matched adults, as a means to maintain forward progression (37). In our study, the faster step time during the arm-rocky analysis may have resulted from our participants incorrectly prioritizing their forward progression over postural stability. This would be in line with previous research demonstrating incorrect task prioritization during walking in this demographic (41).

### Destabilizing Surfaces

In line with our hypothesis, spatiotemporal variability increased, compared to unperturbed walking, when our participants walked on the rocky and rolling hills destabilizing surfaces. However, in contrast to our hypothesis, no differences were found when participants walked on the mediolateral surface compared to unperturbed walking.

Increased spatiotemporal variability was in line with our hypothesis as the destabilizing surfaces mechanically perturbed our participants' gait. However, the COV only measures the magnitude of variability. And while it is a strong predictor of falls in this demographic, it is unable to parse out differences in adaptation vs. motor impairment (6, 17, 33, 34). Indeed, McAndrew et al. discussed that increases in variability can reflect either correct or incorrect foot placement adaptation in response to destabilizing surfaces (23). The authors further went on to discuss that no increases in variability, in response to destabilizing surfaces, would reflect an incorrect response as the neuromuscular system is not correcting foot placement to adapt the base of support at heel-strike to the encountered surface (23). By adapting foot placement, individuals would be accounting for their COM's destabilized trajectory to ensure a stable transfer of the COM between the legs (6, 18, 33, 34). As our sample had a moderate disease progression, it is unlikely that all of the increases in variability were due to incorrect foot placement responses. Rather, increases in spatiotemporal variability may also reflect our participants correctly predicting the destabilization of their COM and appropriately adapting. This would account for lack of findings in the mediolateral-MOS during all the destabilizing surfaces as our participants modified their foot placement to maintain their already existing level of global dynamic stability (18, 42). The ability to adapt to destabilizing surfaces may be linked to partially intact proprioception in people with Parkinson's Disease (43). Indeed, proprioceptive evidence demonstrates that these individuals are still capable of recognizing changes in limb movement and position, but require larger displacement magnitudes, compared to healthy aged matched adults, to do so (43). Thus, it is plausible that the destabilizing surfaces occurred at a magnitude large enough for participants to perceive the destabilization and, at least partially, adapt their foot placement. Further support for this theory is demonstrated by the decreased bilateral average step time, on both the rolling hills and rocky surfaces, as well as the increased average step width on the rocky surface. Current evidence demonstrates that both healthy adults and individuals with lower limb amputations increase walking speed, which would reduce step time, to step off destabilizing surfaces faster and increase step width to widen their mediolateral BOS when traversing destabilizing surfaces (21, 22). Therefore, by our participants executing the correct responses, it indicates that adaptation to destabilizing surfaces is at least partially intact in people with Parkinson's Disease with moderate disease progression. Alternatively, a partially impaired proprioceptive sense may account for the lack of differences between the mediolateral surface and unperturbed walking. Out of all three destabilizing surfaces, the mediolateral surface is the most similar to unperturbed treadmill walking and arguably the least destabilizing condition as the platform only laterally translates. Thus, despite humans being inherently unstable in the mediolateral direction, a larger destabilizing amplitude may have been necessary in order to elicit adaptive stepping responses (5, 23, 44, 45).

Our results hold an important implication for clinicians and researchers to consider. Specifically, therapies should carefully consider terrain type and amplitude when fostering gait adaptation. Particular consideration should be given to the role of variability in reflecting motor adaptation or impairment. Furthermore, clinicians and researchers should carefully consider stage of disease progression when interpreting the COV for fall risk assessment in this demographic.

## Limitations

Several limitations should be considered when interpreting our results. For instance, participants were tested on their optimally medicated state which is demonstrated to affect spatiotemporal variability. Moreover, differences between freezers and non-freezers were not examined. As freezers demonstrate greater postural instability than non-freezers, absent arm swing may have a distinct effect on their dynamic stability. Future research should also consider examining differences between people with Parkinson's Disease and healthy aged matched controls to parse out differences due to age and those that arise due to Parkinson's Disease. Finally, due to our limited sample size, we may have lacked the statistical power to detect all possible interactions of destabilizing surfaces, arm swing conditions and leg asymmetry.

## Conclusion

The current findings on arm swing in all the different analyses suggest that responses to absent arm swing in people with Parkinson's Disease vary depending on the specific terrain encountered. This is expected as evidence examining gait on destabilizing surfaces indicates that compensatory responses are unique to each type of terrain. This in turn would affect how people with Parkinson's Disease respond to absent arm swing on these surfaces. Therefore, rehabilitation therapies should provide diverse environment types that reflect real-world terrains over focused repetitive tasks to foster adaptation to absent arm swing. Further, clinicians should consider interventions that strive to restore the contralateral arm-leg swing pattern. However, based on results in young healthy adults considering which modality to use for restoring arm swing is crucial as explicitly directing individuals to increase arm swing taxes attentional resources and may hold negative consequences on gait in individuals with Parkinson's disease (18). Finally, our results from destabilizing surfaces demonstrate that spatiotemporal variability increases in people with Parkinson's Disease when walking on destabilizing surfaces. While increased variability is a strong predictor of falls, no difference in the MOS suggests that our participants adapted lower limb placement to preserve their already existing global dynamic stability. Adaptation in people with Parkinson's Disease is further corroborated by the reduced average step time and increased step width during the rolling hills and rocky surfaces as these findings mirror responses seen in healthy young adults.

## Data Availability Statement

The raw data supporting the conclusions of this article will be made available by the authors, without undue reservation.

## Ethics Statement

The studies involving human participants were reviewed and approved by the ethics committees of the University of Ottawa and Ottawa General Hospital. The patients/participants provided their written informed consent to participate in this study.

## Author Contributions

JN conceptualized and organized the research project. Data analysis: main analysis (TS), secondary analysis (M-EM) review and critique (JN and M-EM). Statistical analysis: design (TS and JN), execution (TS), review and critique (JN and M-EM). Manuscript: writing of the first draft (TS), review and critique (JN and M-EM). All authors contributed to the article and approved the submitted version.

## Conflict of Interest

The authors declare that the research was conducted in the absence of any commercial or financial relationships that could be construed as a potential conflict of interest.

## References

[1] Stolze H Klebe S Zechlin C Baecker C Friege L Deuschl G. Falls in frequent neurological diseases. J Neurol. (2004) 251:79–84. 10.1007/s00415-004-0276-8 14999493

[2] Bloem BR Grimbergen YAM Cramer M Willemsen M Zwinderman AH. Prospective assessment of falls in Parkinson's disease. J Neurol. (2001) 248:950–8. 10.1007/s004150170047 11757958

[3] Allen NE Schwarzel AK Canning CG. Recurrent falls in Parkinson's disease: a systematic review. Parkinson's Dis. (2013) 2013:1–16. 10.1155/2013/906274 23533953 PMC3606768

[4] Winter DA Human balance and posture control during standing and walking. Gait Post. (1995) 3:193–214. 10.1016/0966-6362(96)82849-9

[5] MacKinnon CD Winter DA. Control of whole body balance in the frontal plane during human walking. J Biomech. (1993) 26:633–44. 10.1016/0021-9290(93)90027-C 8514809

[6] Siragy T Nantel J. Quantifying dynamic balance in young, elderly and Parkinson's individuals: a systematic review. Front Aging Neurosci. (2018) 10:387. 10.3389/fnagi.2018.00387 30524270 PMC6262057

[7] Jehu D Nantel J. Fallers with Parkinson's disease exhibit restrictive trunk control during walking. Gait Post. (2018) 65:246–50. 10.1016/j.gaitpost.2018.07.181 30558939

[8] Plotnik M Giladi N Hausdorff JM. A new measure for quantifying the bilateral coordination of human gait: effects of aging and Parkinson's disease. Exp Brain Res. (2007) 181:561–70. 10.1007/s00221-007-0955-7 17503027

[9] Plotnik M Hausdorff JM. The role of gait rhythmicity and bilateral coordination of stepping in the pathophysiology of freezing of gait in Parkinson's disease. Mov Disord. (2008) 23:S444–50. 10.1002/mds.21984 18668626

[10] Hausdorff JM Cudkowicz ME Firtion R Wei JY Goldberger AL. Gait variability and basal ganglia disorders: stride-to-stride variations of gait cycle timing in Parkinson's disease and Huntington's disease. Mov Disord. (1998) 13:428–37. 10.1002/mds.870130310 9613733

[11] Galna B Murphy AT Morris ME. Obstacle crossing in Parkinson's disease: mediolateral sway of the centre of mass during level-ground walking and obstacle crossing. Gait Posture. (2013) 38:790–4. 10.1016/j.gaitpost.2013.03.024 23647655

[12] Adkin AL Bloem BR Allum JHJ. Trunk sway measurements during stance and gait tasks in Parkinson's disease. Gait Post. (2005) 22:240–9. 10.1016/j.gaitpost.2004.09.009 16278966

[13] Greenspan SL Myers ER Kiel DP Parker RA Hayes WC Resnick NM. Fall direction, bone mineral density, and function: risk factors for hip fracture in frail nursing home elderly. Am J Med. (1998) 104:539–45. 10.1016/S0002-9343(98)00115-6 9674716

[14] Greenspan S Resnick N. ‘Senile’ Osteoporosis Reconsidered. JAMA Network. (1989) 261:1025–1029. 10.1001/jama.1989.03420070075034 2644455

[15] Marigold DS Misiaszek JE. Whole-body responses: neural control and implications for rehabilitation and fall prevention. Neuroscientist. (2009) 15:36–46. 10.1177/1073858408322674 19218229

[16] Meyns P Bruijn SM Duysens J. The how and why of arm swing during human walking. Gait Posture. (2013) 38:555–62. 10.1016/j.gaitpost.2013.02.006 23489950

[17] Siragy T Nantel J. Absent arm swing and dual tasking decreases trunk postural control and dynamic balance in people with Parkinson's disease. Front Neurol. (2020) 11:213. 10.3389/fneur.2020.00213 32362863 PMC7180219

[18] Siragy T Mezher C Hill A Nantel J. Active arm swing and asymmetric walking leads to increased variability in trunk kinematics in young adults. J Biomech. (2020) 99:109529. 10.1016/j.jbiomech.2019.109529 31839359

[19] Hill A Nantel J. The effects of arm swing amplitude and lower-limb asymmetry on gait stability. PLoS ONE. (2019) 14:e0218644. 10.1371/journal.pone.0218644 31860669 PMC6924645

[20] Bruijn SM Meijer OG Beek PJ van Dieen JH. The effects of arm swing on human gait stability. J Exp Biol. (2010) 213:3945–52. 10.1242/jeb.045112 21075935

[21] Sturk JA Lemaire ED Sinitski EH Dudek NL Besemann M Hebert JS. Maintaining stable transfemoral amputee gait on level, sloped and simulated uneven conditions in a virtual environment. Disabil Rehabi. (2019) 14:226–35. 10.1080/17483107.2017.1420250 29276850

[22] Sinitski EH Lemaire ED Baddour N Besemann M Dudek NL Hebert JS. Fixed and self-paced treadmill walking for able-bodied and transtibial amputees in a multi-terrain virtual environment. Gait Post. (2015) 41:568–73. 10.1016/j.gaitpost.2014.12.016 25661003

[23] McAndrew PM Dingwell JB Wilken JM. Walking variability during continuous pseudo-random oscillations of the support surface and visual field. J Biomech. (2010) 43:1470–5. 10.1016/j.jbiomech.2010.02.003 20346453 PMC2866814

[24] McAndrew PM Wilken JM Dingwell JB. Dynamic stability of human walking in visually and mechanically destabilizing environments. J Biomech. (2011) 44:644–9. 10.1016/j.jbiomech.2010.11.007 21094944 PMC3042508

[25] Mirelman A Bernad-Elazari H Thaler A Giladi-Yacobi E Gurevich T Gana-Weisz M. Arm swing as a potential new prodromal marker of Parkinson's disease: arm swing as a new prodromal marker of PD. Mov Disord. (2016) 31:1527–34. 10.1002/mds.26720 27430880 PMC5053872

[26] Goetz CG Poewe W Rascol O Sampaio C Stebbins GT Counsell C. Movement disorder society task force report on the hoehn and yahr staging scale: status and recommendations the *movement* disorder society task force on rating scales for Parkinson's disease. Mov Disord. (2004) 19:1020–8. 10.1002/mds.20213 15372591

[27] Movement Disorder Society Task Force on Rating Scales for Parkinson's Disease. The Unified Parkinson's Disease Rating Scale (UPDRS): Status and recommendations. Mov Disord. (2003) 18:738–750. 10.1002/mds.10473 12815652

[28] Hof AL. The ‘extrapolated center of mass’ concept suggests a simple control of balance in walking. Hum Mov Sci. (2008) 27:112–25. 10.1016/j.humov.2007.08.003 17935808

[29] Hof AL Gazendam MGJ Sinke WE. The condition for dynamic stability. J Biomech. (2005) 38:1–8. 10.1016/j.jbiomech.2004.03.025 15519333

[30] Hak L Houdijk H Steenbrink F Mert A van der Wurff P Beek PJ. Stepping strategies for regulating gait adaptability and stability. J Biomech. (2013) 46:905–11. 10.1016/j.jbiomech.2012.12.017 23332822

[31] Bruijn SM Meijer OG Beek PJ van Dieen JH. Assessing the stability of human locomotion: a review of current measures. J Roy Soc Interf. (2013) 10:20120999–20120999. 10.1098/rsif.2012.0999 23516062 PMC3645408

[32] Huang Y Meijer OG Lin J Bruijn SM Wu W Lin X. The effects of stride length and stride frequency on trunk coordination in human walking. Gait Post. (2010) 31:444–9. 10.1016/j.gaitpost.2010.01.019 20171890

[33] Hausdorff JM. Gait dynamics in Parkinson's disease: common and distinct behavior among stride length, gait variability, and fractal-like scaling. Chaos Interdisc J Nonlinear Sci. (2009) 19:026113. 10.1063/1.3147408 19566273 PMC2719464

[34] Hausdorff JM. Gait variability: methods, modeling and meaning. J Neuroeng Rehabil. (2005) 2:19. 10.1186/1743-0003-2-19 16033650 PMC1185560

[35] Poewe W Seppi K Tanner CM Halliday GM Brundin P Volkmann J Parkinson disease. Nat Rev Dis Primers. (2017) 3:17013 10.1038/nrdp.2017.13 28332488

[36] Blandini F Nappi G Tassorelli C Martignoni E. Functional changes of the basal ganglia circuitry in Parkinson's disease. Prog Neurobiol. (2000) 62:63–88. 10.1016/S0301-0082(99)00067-2 10821982

[37] Morris ME Iansek R Matyas TA Summers JJ. Stride length regulation in Parkinson's disease: normalization strategies and underlying mechanisms. Brain. (1996) 119:551–68. 10.1093/brain/119.2.551 8800948

[38] Djaldetti R Ziv I Melamed E. The mystery of motor asymmetry in Parkinson's disease. Lancet Neurol. (2006) 5:796–802. 10.1016/S1474-4422(06)70549-X 16914408

[39] Louie S Koop MM Frenklach A Bronte-Stewart H. Quantitative lateralized measures of bradykinesia at different stages of Parkinson's disease: the role of the less affected side. Mov Disord. (2009) 24:1991–7. 10.1002/mds.22741 19672996

[40] Herman T Giladi N Gurevich T Hausdorff JM. Gait instability and fractal dynamics of older adults with a “cautious” gait: why do certain older adults walk fearfully? Gait Post. (2005) 21:178–85. 10.1016/j.gaitpost.2004.01.014 15639397

[41] Yogev-Seligmann G Hausdorff JM Giladi N. The role of executive function and attention in gait. Mov Disord. (2008) 23:329–42. 10.1002/mds.21720 18058946 PMC2535903

[42] Rosenblatt NJ Grabiner MD. Measures of frontal plane stability during treadmill and overground walking. Gait Post. (2010) 31:380–4. 10.1016/j.gaitpost.2010.01.002 20129786

[43] Konczak J Corcos DM Horak F Poizner H Shapiro M Tuite P. Proprioception and motor control in Parkinson's disease. J Motor Behav. (2009) 41:543–52. 10.3200/35-09-002 19592360

[44] Kuo AD Stabilization of lateral motion in passive dynamic walking. Int J Robot Res. (1999) 18:917–30. 10.1177/02783649922066655

[45] Bauby CE Kuo AD. Active control of lateral balance in human walking. J Biomech. (2000) 33:1433–40. 10.1016/S0021-9290(00)00101-9 10940402

